# Highly Pathogenic Avian Influenza A(H5N8) Virus, Cameroon, 2017

**DOI:** 10.3201/eid2407.172120

**Published:** 2018-07

**Authors:** Abel Wade, Simon Dickmu Jumbo, Bianca Zecchin, Alice Fusaro, Taiga Taiga, Alice Bianco, Poueme N. Rodrigue, Angela Salomoni, Jean Marc Feussom Kameni, Gianpiero Zamperin, Robert Nenkam, Yacouba Foupouapouognigni, Souley Abdoulkadiri, Yaya Aboubakar, Lidewij Wiersma, Giovanni Cattoli, Isabella Monne

**Affiliations:** Laboratoire National Veterinaire, Yaoundé and Garoua, Cameroon (A. Wade, S. Dickmu Jumbo, P.N. Rodrigue, R. Nenkam, Y. Foupouapouognigni, S. Abdoulkadiri, Y. Aboubakar);; Ministry of Livestock, Fisheries and Animal Industries, Yaoundé (A. Wade, T. Taiga, J.M. Feussom Kameni);; Istituto Zooprofilattico Sperimentale delle Venezie, Padova, Italy (B. Zecchin, A. Fusaro, A. Bianco, A. Salomoni, G. Zamperin, I. Monne);; Food and Agriculture Organization of the United Nations, Rome, Italy (L. Wiersma);; Animal Production and Health Laboratory, Seibersdorf, Austria (G. Cattoli)

**Keywords:** highly pathogenic avian influenza virus, H5N8, Cameroon, clade 2.3.4.4, influenza, viruses

## Abstract

Highly pathogenic avian influenza A(H5N8) viruses of clade 2.3.4.4 spread into West Africa in late 2016 during the autumn bird migration. Genetic characterization of the complete genome of these viruses detected in wild and domestic birds in Cameroon in January 2017 demonstrated the occurrence of multiple virus introductions.

Since the first detection in China in 1996, highly pathogenic avian influenza (HPAI) viruses of the H5 subtype descendent of the H5N1 virus A/goose/Guangdong/1/1996 (Gs/GD/96) have evolved into multiple genetic clades (*1**,**2*) that have been threatening poultry worldwide. Since 2010, clade 2.3.4 has demonstrated an unusual propensity to replace its N1 subtype and acquire different neuraminidase (NA) genes from unrelated avian influenza viruses through reassortment, which has resulted in the emergence of new viral subtypes within the Gs/GD/96 H5 lineage (e.g., N2, N5, N6, N8). In late spring 2016, reassortant HPAI A(H5N8) clade 2.3.4.4 (group B) virus was detected in migratory wild birds in Qinghai Lake, China (*3*), and in the salt lake system of Uvs Nuur, on the border between Mongolia and the Russian Federation (*4*). Since then, this newly emerged virus has caused multiple outbreaks of the disease in poultry and wild birds across Europe, Asia, and the Middle East and has extended not only to countries in northern, central, and western Africa, as did the previous Gs/GD/96 H5 lineage outbreaks, but also to the eastern and southern parts of Africa (*5*). We investigated the epidemiology of the outbreaks in early 2017 of HPAI H5N8 virus in domestic and wild birds in Cameroon and determined the viruses’ possible origin of this virus through whole-genome analyses.

## The Study

On January 2, 2017, high death rates were reported in Indian peafowl (*Pavo cristatus*) in a backyard exotic poultry farm located in the town of Makilingaye (Tokombéré district of Mayo-Sava division), a village neighboring Nigeria in the far-north region of Cameroon. Samples were collected by the Cameroon Epidemio-Surveillance Network (Reseau d’Epidemio-Surveillance au Cameroun [RESCAM]) of the Ministry of Livestock, Fisheries and Animal Industries and were sent to the National Veterinary Laboratory (LANAVET) in Garoua, where the H5N8 subtype was diagnosed. Almost all the peafowl (103/107) died within ≈2 weeks. Death in chickens (*Gallus gallus domesticus*, 24/24) housed in the same compound was delayed and appeared 5 days later than in peafowl. Following the laboratory confirmation of the first outbreak, the Cameroon government, through the Ministry of Livestock, Fisheries and Animal Industries, implemented prompt and strong control measures to stop the spread of the virus and reduce the risk of human infections. Stamping out was deployed together with movement restrictions and virological surveillance; disinfection of premises and contact materials was intensified. The RESCAM team conducted a routine avian influenza survey in the Maroua, Yagoua, and Guidiguis central poultry markets in the far-north region. All the samples were analyzed at LANAVET Garoua and Annex Yaounde; H5N8 virus was detected in 5 birds (1 pigeon, 1 chicken, 2 guinea fowls, and 1 duck) out of 122 birds.

We sequenced the hemagglutinin (HA) and NA gene segments of the virus A/Indian peafowl/Cameroon/17RS1661-6/2017, identified from an Indian peafowl in Makilingaye, at the Istituto Zooprofilattico Sperimentale delle Venezie in Legnaro, Italy, along with the complete genomes of 2 positive samples collected from domestic birds (chicken and duck) and the partial genome (all segments except for polymerase acidic protein [PA] and polymerase basic protein 1 [PB1]) of a sample from a wild pigeon, all identified in the Maroua and Yagoua markets (Table). A detailed description of the methods used for sequencing and genetic analyses is provided in Technical Appendix 1 and details on the HA gene segments used for the analysis are given in Technical Appendix 2. We submitted consensus sequences to GenBank (accession nos. MG650618–41).

**Table:** Epidemiologic information of viruses characterized in study of highly pathogenic avian influenza A(H5N8) virus, Cameroon, January 2017*

Virus	Sample type	Location	EpiFlu accession numbers for 8 gene segments
A/chicken/Cameroon/17RS1661-1/2017	Tracheal swab	Maroua market	HA, MG650619; MP, MG650622; NA, MG650626; NP, MG650630; NS, MG650632; PA, MG650635; PB1, MG650638; PB2, MG650641
A/duck/Cameroon/17RS1661-3/2017	Tracheal swab	Yagoua market	HA, MG650620; MP, MG650623; NA, MG650627; NP,MG650629; NS, MG650633; PA, MG650636; PB1, MG650637; PB2, MG650639
A/pigeon/Cameroon/17RS1661-4/2017	Cloacal swab	Maroua market	HA, MG650621; MP, MG650624; NA, MG650628; NP, MG650631; NS, MG650634; PA, NR; PB1, na; PB2, MG650640
A/Indian peafowl/Cameroon/17RS1661-6/2017	Tracheal swab	Makilingaye	HA, MG650619; MP, NR; NA, MG650626; NP, NR; NS, NR; PA, NR; PB1, NR; PB2, NR

*HA, hemagglutinin; MP, matrix protein, NA, neuraminidase; NR, not reported; NP, nucleoprotein; NS, nonstructural proteins; PA, polymerase acidic protein; PB1, polymerase basic protein 1; PB2, polymerase basic protein 2. †From GISAID EpiFlu database (online Technical Appendix 2, https://wwwnc.cdc.gov/EID/article/24/7/17-2120-Techapp2.xlsx).

Topology of the phylogenetic tree based on the HA gene segment showed that the 4 H5N8 viruses detected in Cameroon in 2017 fell within genetic clade 2.3.4.4 group B (Figure; Technical Appendix 1 Figure 1). The HA gene segments of the 3 viruses from Makilingaye (A/Indian peafowl/Cameroon/17RS1661–6/2017) and Maroua market clustered together; similarity ranged from 99.5% (between the viruses collected from Makilingaye and Maroua markets) to 100% (between the 2 viruses from Maroua market). These segments grouped with H5N8 viruses collected during 2016–2017 in South Korea, Egypt, Italy, Siberia, and India (similarity between 99.3% and 99.6%). However, the virus A/duck/Cameroon/17RS1661-3/2017 identified in Yagoua market clustered separately and showed the highest similarity (99.3%) to a virus from India (A/duck/India/10CA01/2016) (Figure).

**Figure:**
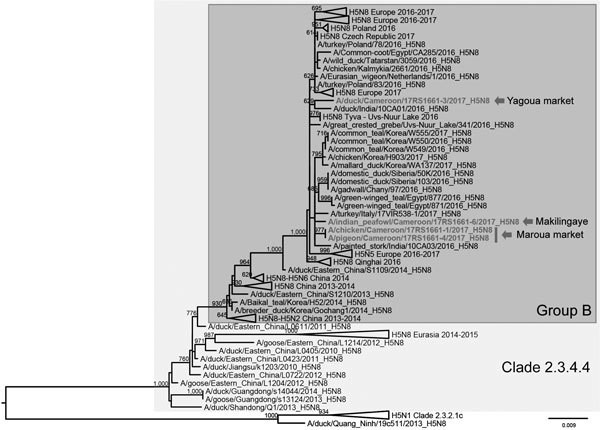
Maximum-likelihood phylogenetic tree of the HA gene of highly pathogenic avian influenza A(H5N8) virus from Cameroon (bold) and reference viruses. Arrows indicate location where Cameroon viruses were obtained. Bootstrap supports >600/1,000 are indicated next to the nodes. Scale bar indicates numbers of nucleotide substitutions per site.

We confirmed the different clustering of the viruses collected from distinct markets in Cameroon by the analyses of the other gene segments, which suggests the occurrence of >2 distinct viral introductions in the country. Specifically, sequences of the 2 viruses from Maroua market were identical for all the available gene segments (Table) and clustered with viruses collected in Asia, Europe, and Egypt (Technical Appendix 1 Figures 2–8). On the other hand, the virus A/duck/Cameroon/17RS1661-3/2017 grouped with the A/duck/India/10CA01/2016 virus (similarity 94.3–99.9%), except for the nucleoprotein (NP) gene, where it showed the highest identity (99.3%) with a virus collected in 2016 in the Russian Federation (Uvs Nuur Lake). The lack of genetic information on other H5N8 viruses detected in Africa makes it impossible to pinpoint how these viruses entered Cameroon and spread, nor is it feasible to determine where the 2 introductions occurred. The time to the most recent common ancestor estimated for the HA gene (Technical Appendix 1) suggested that 2 introductions into Africa may have occurred almost simultaneously during March–December 2016 (Technical Appendix 1 Figure 9). However, analyses of the amino acid sequences show that A/duck/Cameroon/17RS1661-3/2017 possesses the N319K mutation in the NP protein, which has been reported to enhance polymerase activity and stimulate vRNA synthesis in mammalian cells (*6*). In addition, A/chicken/Cameroon/17RS1661-1/2017 contains the V100A mutation in the PA protein, which is an amino acid signature typical of human influenza viruses (*7*).

## Conclusions

Nigeria was the first country in West Africa to report the presence of the HPAI H5N8 virus in November 2016 (*8**,**9*). Less than 2 months later, in January 2017, the virus was detected in the far-north region of Cameroon in Makilingaye, close to the Nigerian border. Considering the extensive and porous frontier between Cameroon and Nigeria, trade and movement of poultry might have played a key role in the spread of the virus. However, the involvement of wild birds cannot be excluded. The region contains several wetlands (Domayo River, Mayo Kani River, and Maga Lake) where different wild birds congregate, in particular during the dry season (December–April), when the virus was first detected. The almost simultaneous detection (early January 2017) of the H5N8 virus in poultry and wild birds in distant locations in Africa, such as Tunisia and Uganda, suggests that the role of wild birds in the introduction and/or dissemination of the virus in the region should not be overlooked. Of note, the first outbreak caused by the Gs/GD/96 H5 lineage in Cameroon, in 2006, also occurred in the far-north region of Cameroon at about the same time. This finding might suggest a common pathway for introduction of the virus into this area and highlights the need to improve surveillance in this region.

Although the epidemiologic and genetic data are insufficient to establish definite pathways and time of introduction of H5N8 virus into West Africa, this study demonstrates that >2 distinct H5N8 viruses entered Cameroon. This finding, together with the evidence that this event represents the third incursion of a Gs/GD/96-lineage H5 HPAI virus into Cameroon, again underlines the need to perform avian influenza surveillance on an ongoing basis for rapid identification and response to outbreaks in this area.

Technical Appendix 1 Details on materials and methods used in the study of influenza A(H5N8) virus, Cameroon, 2017.

Technical Appendix 2 Hemagglutinin (HA) gene segments of influenza virus strains used for analysis of influenza A(H5N8) virus, Cameroon, 2017.
