# Supplementary material for: Highly Pathogenic Avian Influenza A(H5N8) Virus, Cameroon, 2017
Source: Emerg Infect Dis. 2018 Jul;24(7):1367–70. doi: 10.3201/eid2407.172120 (PMC6038759; doi:10.3201/eid2407.172120)
Supplement: Technical Appendix 1 — Details on materials and methods used in the study of influenza A(H5N8) virus, Cameroon, 2017. [file 17-2120-Techapp-s1.pdf]

Article DOI: https://doi.org/10.3201/eid2407.172120

Highly Pathogenic Avian Influenza A(H5N8) Virus, Cameroon, 2017

Technical Appendix

Materials and Methods

Sample Collection and Laboratory Analysis

The Cameroon Epidemio-Surveillance Network (RESCAM) of the Ministry of Livestock, Fisheries and Animal Industries (MINEPIA) collected 1 dead peacock during the outbreak in Makilingaye township and sent it to the National Veterinary Laboratory (LANAVET) in Garoua for analysis. In addition, we collected tracheal and cloacal swabs from 50 chickens, 55 ducks, 11 guinea fowls, and 6 pigeons during avian influenza field investigations in Maroua, Yagoua, and Guidiguis' central poultry markets (Technical Appendix Table 1). We sent these samples to the same laboratory for analysis. We extracted viral RNA from all the samples using the QIamp RNA mini kit (QIAGEN, Hilden, Germany) according to the manufacturer's protocol. We conducted RNA detection of the H5 gene using real-time reverse transcription PCR (RT-PCR) (*1*) followed by RNA detection of the N8 gene (*2*).

Genome Sequencing

At the World Organization for Animal Health (OIE)/Food and Agriculture Organization of the United Nations (UN-FAO) Reference Laboratory for Avian Influenza (Istituto Zooprofilattico Sperimentale delle Venezie, Legnaro, PD) in Italy, total RNA was purified from 4 HPAI H5N8-positive clinical samples using the Nucleospin RNA kit (Macherey–Nagel, Düren, Germany). Complete influenza A virus genomes were amplified with the SuperScript III One-Step RT-PCR system with Platinum Taq High Fidelity (Invitrogen, Carlsbad, CA, USA) using 1 pair of primers complementary to the conserved elements of the influenza A virus promoter (*3*). Sequencing libraries were obtained using a Nextera DNA XT Sample preparation kit (Illumina, San Diego, CA, USA) following the manufacturer's instructions and quantified using the Qubit dsDNA High Sensitivity kit (Invitrogen). We determined the average fragment

length using the Agilent High Sensitivity Bioanalyzer Kit (Agilent Technologies, Santa Clara, CA, USA). The indexed libraries were pooled in equimolar concentrations and sequenced in multiplex for 250 bp paired-end on Illumina MiSeq, according to the manufacturer's instructions.

High-Throughput Sequencing Data Analysis

We assessed the Illumina reads' quality using FastQC v0.11.2 (https://www.bioinformatics.babraham.ac.uk/projects/fastqc). We filtered the raw data by removing reads with more than 10% of undetermined (N) bases, reads with more than 100 bases with Q score below 7, and duplicated paired-end reads. We clipped the remaining reads from Illumina Nextera XT adaptors with scythe v0.991 (https://github.com/vsbuffalo/scythe) and trimmed them with sickle v1.33 (https://github.com/najoshi/sickle). We discarded reads shorter than 80 bases or unpaired after previous filters. We then aligned the high-quality reads against a reference genome using BWA v0.7.12 (*4*). We processed the alignments with Picard-tools v2.1.0 (http://picard.sourceforge.net) and GATK v3.5 (*5,6,7*) to correct potential errors, realign reads around indels, and recalibrate base quality. We called single nucleotide polymorphisms using LoFreq v2.1.2 (*8*) and used the outputs to generate the consensus sequences. We obtained the complete genome of viruses A/chicken/Cameroon/17RS1661–1/2017 and A/duck/Cameroon/17RS1661–3/2017; the HA, NA, MA, NP, NS, and PB2 gene segments of A/pigeon/Cameroon/17RS1661–4/2017; and the HA and NA gene segments of the virus A/Indian_peafowl/Cameroon/17RS1661–6/2017.

Phylogenetic Analyses

We compared the sequences of the 8 gene segments of the H5N8 viruses from Cameroon with all the H5 sequences belonging to clade 2.3.4.4 group B available in GISAD and with the 50 most related sequences resulted from the BLAST search. We aligned sequences of each gene segment using MAFFT v. 7 (*9*). We obtained maximum likelihood phylogenetic trees for each gene segment using the best-fit general time-reversible model of nucleotide substitution with gamma-distributed rate variation among sites (with 4 rate categories, Γ4) and a heuristic SPR branch-swapping search (*10*) available in the PhyML program v3.1. To assess the robustness of individual nodes of the phylogeny, we performed 1,000 bootstrap replicates. Phylogenetic trees were visualized with the program FigTree v1.4.2 (http://tree.bio.ed.ac.uk/software/figtree/).

Estimation of Time to the Most Recent Common Ancestor

We estimated the tMRCAs of the HPAI H5N8 identified in Cameroon for the HA gene segment using BEAST v1.8.4 software (*11*). We selected a HKY85 + Γ_4 nucleotide substitution model with two data partitions reflecting codon positions (1st + 2nd positions, 3rd position) and with base frequency unlinked across all codon positions (SRD06 substitution model). In addition, we used a relaxed (uncorrelated lognormal) molecular clock and constant population size coalescent as the tree prior. We used Markov chain Monte Carlo (MCMC) and chain lengths of 100 million iterations to achieve convergence as assessed using Tracer v1.6 (http://beast.bio.ed.ac.uk/Tracer). The maximum clade credibility (MCC) phylogenetic tree was summarized from the posterior distribution of trees using TreeAnnotator v1.8.4 (*11*) after the removal of an appropriate burn-in (10% of the samples). The MCC tree was visualized using FigTree v1.4.2 (http://tree.bio.ed.ac.uk/software/figtree/) (Technical Appendix Figure 9).

References

1. Monne I, Ormelli S, Salviato A, De Battisti C, Bettini F, Salomoni A, et al. Development and validation of a one-step real-time PCR assay for simultaneous detection of subtype H5, H7, and H9 avian influenza viruses. J Clin Microbiol. 2008;46:1769–73. PubMed http://dx.doi.org/10.1128/JCM.02204-07

2. Fereidouni SR, Starick E, Grund C, Globig A, Mettenleiter TC, Beer M, et al. Rapid molecular subtyping by reverse transcription polymerase chain reaction of the neuraminidase gene of avian influenza A viruses. Vet Microbiol. 2009;135:253–60. PubMed http://dx.doi.org/10.1016/j.vetmic.2008.09.077

3. Zhou B, Donnelly ME, Scholes DT, St. George K, Hatta M, Kawaoka Y, et al. Single-reaction genomic amplification accelerates sequencing and vaccine production for classical and swine origin human influenza A viruses. J Virol. 2009;83:10309–13. PubMed http://dx.doi.org/10.1128/JVI.01109-09

4. Li H, Durbin R. Fast and accurate long-read alignment with Burrows-Wheeler transform. Bioinformatics. 2010;26:589–95. PubMed http://dx.doi.org/10.1093/bioinformatics/btp698

5. McKenna A, Hanna M, Banks E, Sivachenko A, Cibulskis K, Kernytsky A, et al. The Genome Analysis Toolkit: a MapReduce framework for analyzing next-generation DNA sequencing data. Genome Res. 2010;20:1297–303. PubMed http://dx.doi.org/10.1101/gr.107524.110

6. DePristo MA, Banks E, Poplin R, Garimella KV, Maguire JR, Hartl C, et al. A framework for variation discovery and genotyping using next-generation DNA sequencing data. Nat Genet. 2011;43:491–8. PubMed http://dx.doi.org/10.1038/ng.806

7. Van der Auwera GA, Carneiro MO, Hartl C, Poplin R, Del Angel G, Levy-Moonshine A, et al. From FastQ data to high confidence variant calls: the Genome Analysis Toolkit best practices pipeline. Curr Protoc Bioinformatics. 2013;43:11.10.1–33. http://dx.doi.org/10.1002/0471250953.bi1110s43

8. Wilm A, Aw PP, Bertrand D, Yeo GH, Ong SH, Wong CH, et al. LoFreq: a sequence-quality aware, ultra-sensitive variant caller for uncovering cell-population heterogeneity from high-throughput sequencing datasets. Nucleic Acids Res. 2012;40:11189–201. PubMed http://dx.doi.org/10.1093/nar/gks918

9. Katoh K, Standley DM. MAFFT multiple sequence alignment software version 7: improvements in performance and usability. Mol Biol Evol. 2013;30:772–80. PubMed http://dx.doi.org/10.1093/molbev/mst010

10. Guindon S, Gascuel O. A simple, fast, and accurate algorithm to estimate large phylogenies by maximum likelihood. Syst Biol. 2003;52:696–704. PubMed http://dx.doi.org/10.1080/10635150390235520

11. Drummond AJ, Rambaut A. BEAST: Bayesian evolutionary analysis by sampling trees. BMC Evol Biol. 2007;7:214. PubMed http://dx.doi.org/10.1186/1471-2148-7-214

Technical Appendix 1 Table. Number of positive samples in the total number of sampled animals (no. pos/no. samples) from each market

Animal	Maroua market no. pos/no. samples	Yagoua market no. pos/no. samples	Guidiguis market no. pos/no. samples	Total no. pos/no. samples
Chicken	1/13	0/26	0/11	1/50
Pigeon	1/4	0/1	0/1	1/6
Guinea fowl	0/4	2/4	0/3	2/11
Duck	0/16	1/24	0/15	1/55
Total	2/37	3/55	0/30	5/122

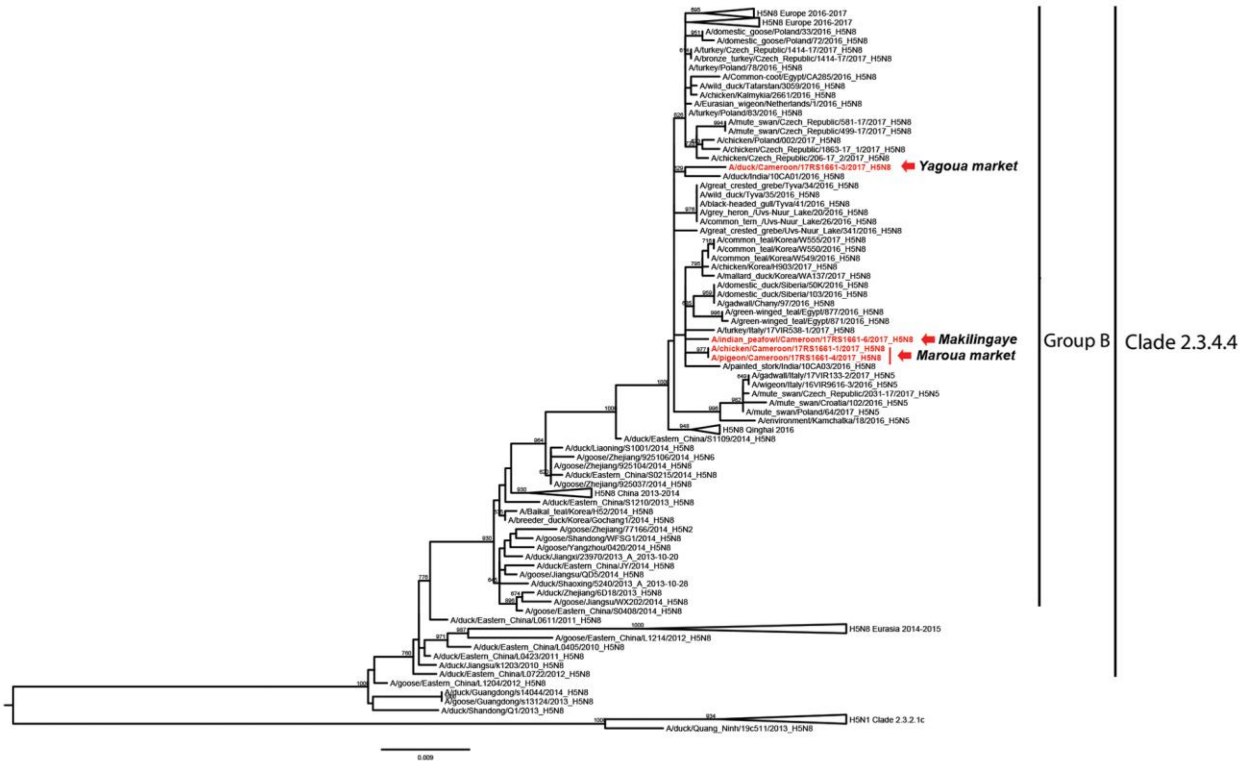

Technical Appendix 1 Figure 1. Maximum likelihood phylogenetic tree of the hemagglutinin (HA) gene. The H5N8 viruses from Cameroon are marked in red. Bootstrap supports higher than 600/1000 are indicated next to the nodes; scale bar indicates numbers of nucleotide substitutions per site.

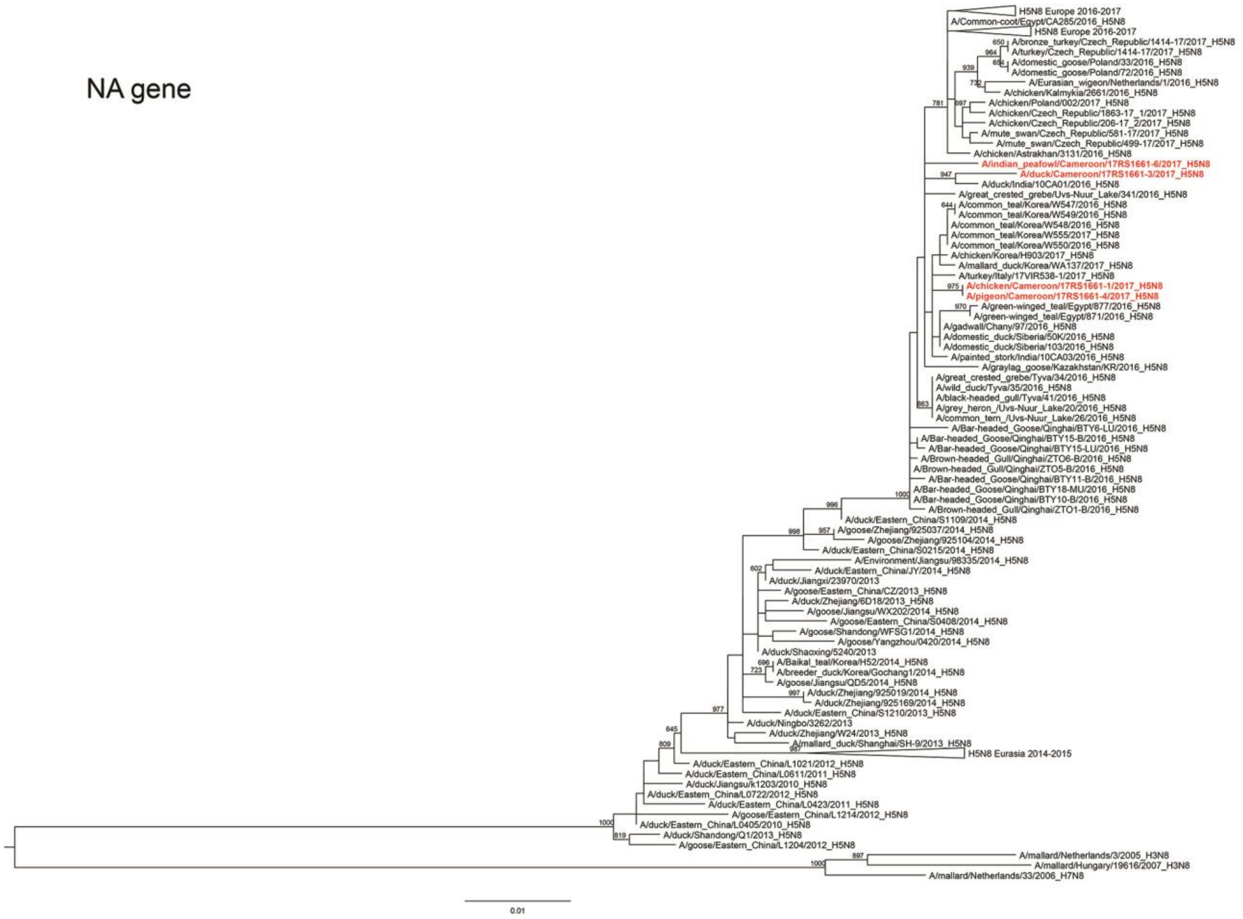

Technical Appendix 1 Figure 2. Maximum likelihood phylogenetic tree of the neuraminidase (NA) gene. The H5N8 viruses from Cameroon are marked in red. Bootstrap supports higher than 600/1000 are indicated next to the nodes; scale bar indicates numbers of nucleotide substitutions per site.

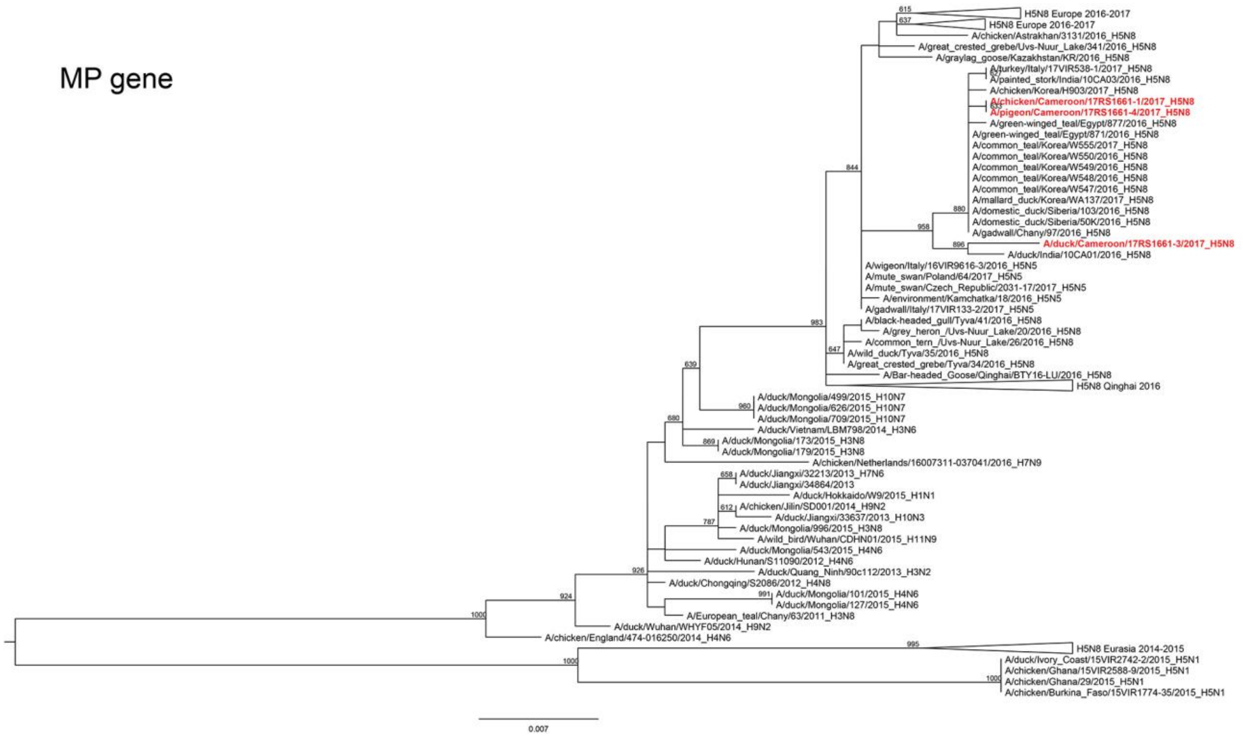

Technical Appendix 1 Figure 3. Maximum likelihood phylogenetic tree of the matrix protein (MP) gene. The H5N8 viruses from Cameroon are marked in red. Bootstrap supports higher than 600/1000 are indicated next to the nodes; scale bar indicates numbers of nucleotide substitutions per site.

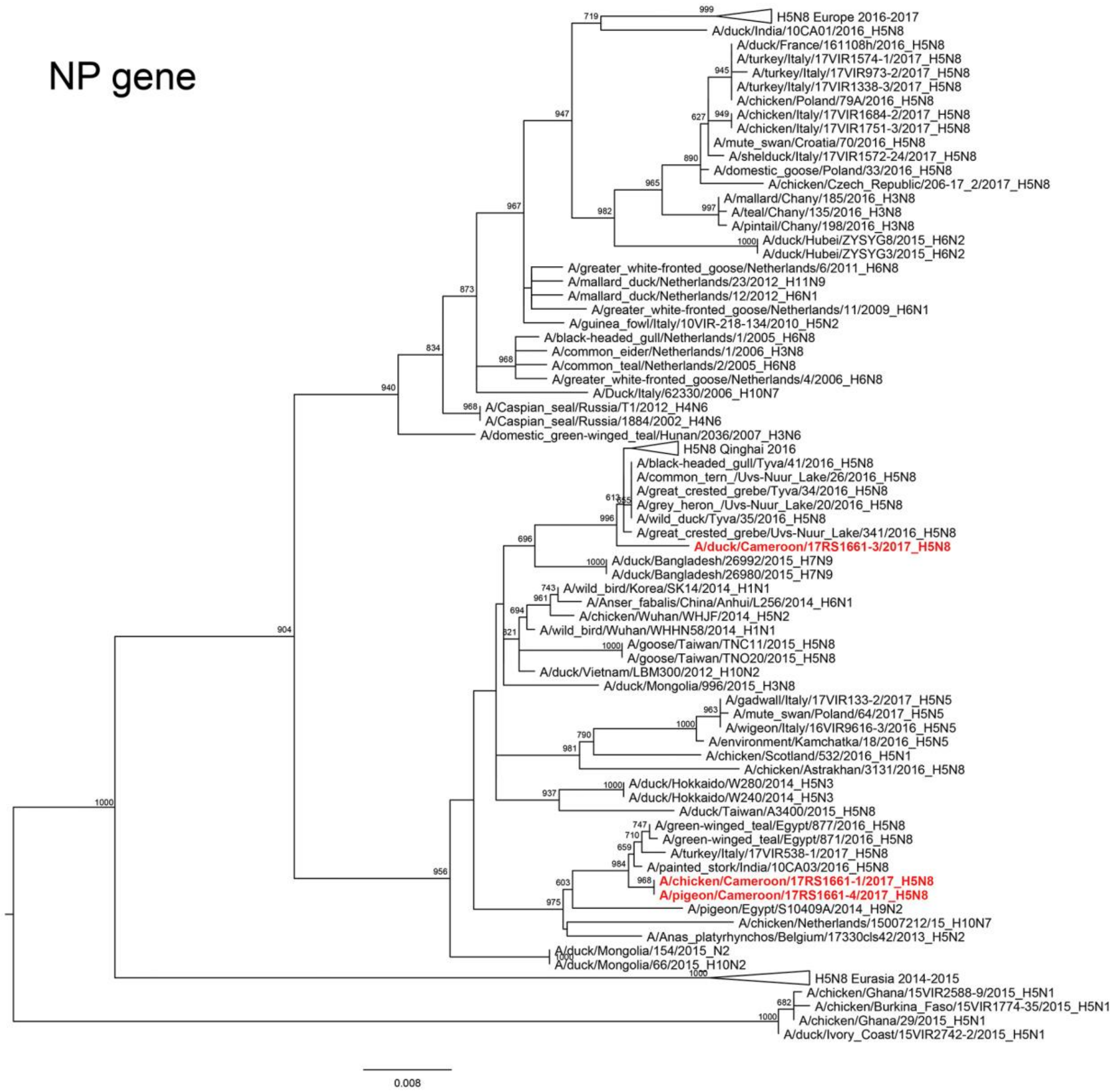

Technical Appendix 1 Figure 4. Maximum likelihood phylogenetic tree of the nucleoprotein (NP) gene. The H5N8 viruses from Cameroon are marked in red. Bootstrap supports higher than 600/1000 are indicated next to the nodes; scale bar indicates numbers of nucleotide substitutions per site.

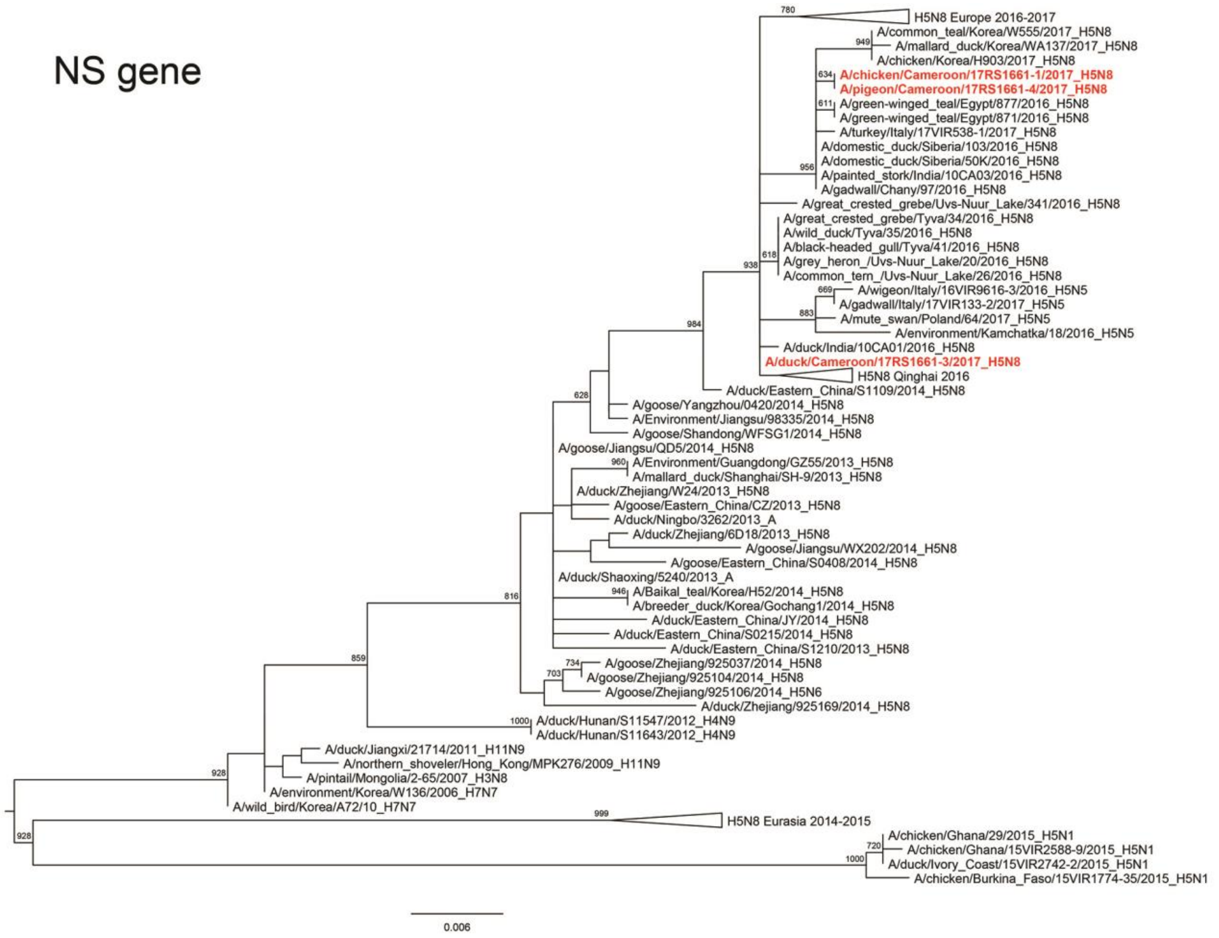

Technical Appendix 1 Figure 5. Maximum likelihood phylogenetic tree of the nonstructural protein (NS) gene. The H5N8 viruses from Cameroon are marked in red. Bootstrap supports higher than 600/1000 are indicated next to the nodes; scale bar indicates numbers of nucleotide substitutions per site.

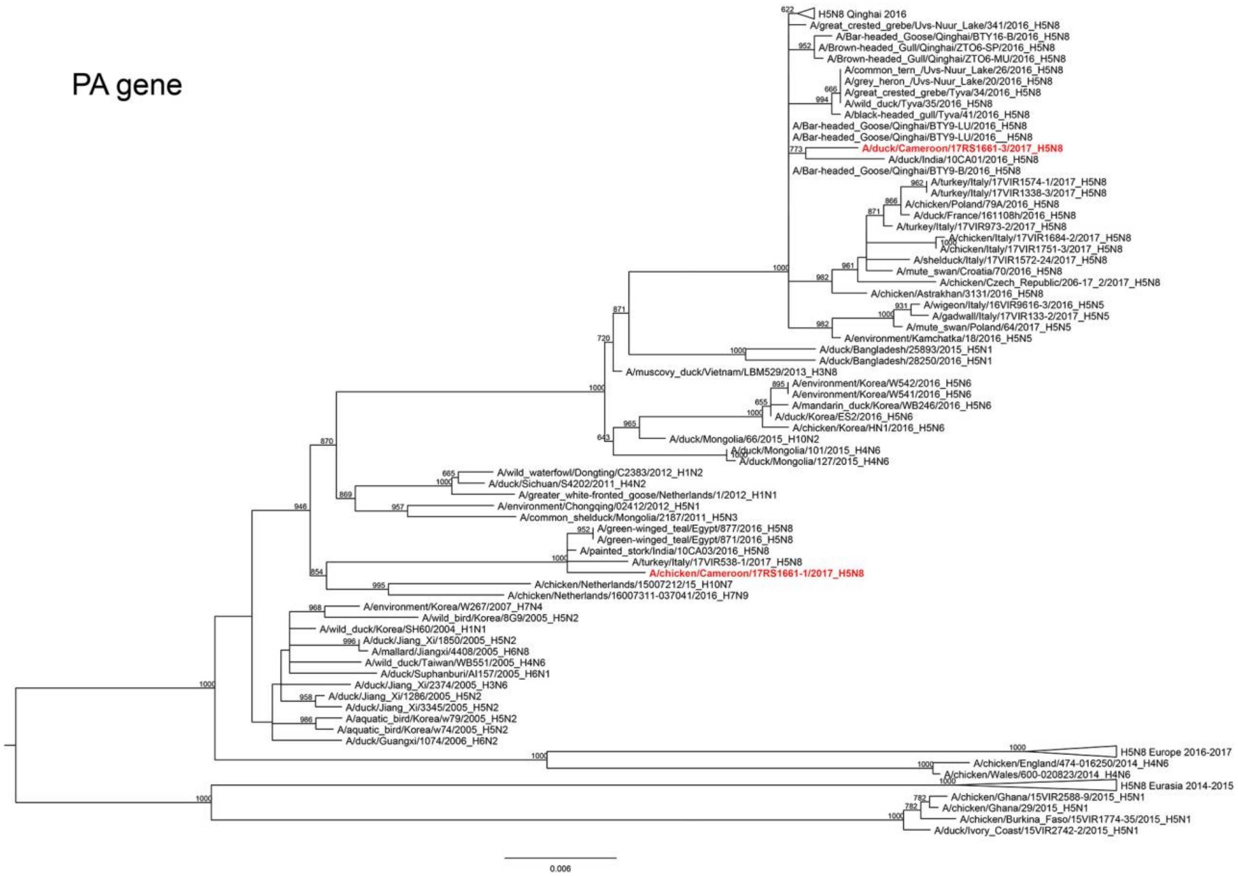

Technical Appendix 1 Figure 6. Maximum likelihood phylogenetic tree of the polymerase acidic protein (PA) gene. The H5N8 viruses from Cameroon are marked in red. Bootstrap supports higher than 600/1000 are indicated next to the nodes; scale bar indicates numbers of nucleotide substitutions per site.

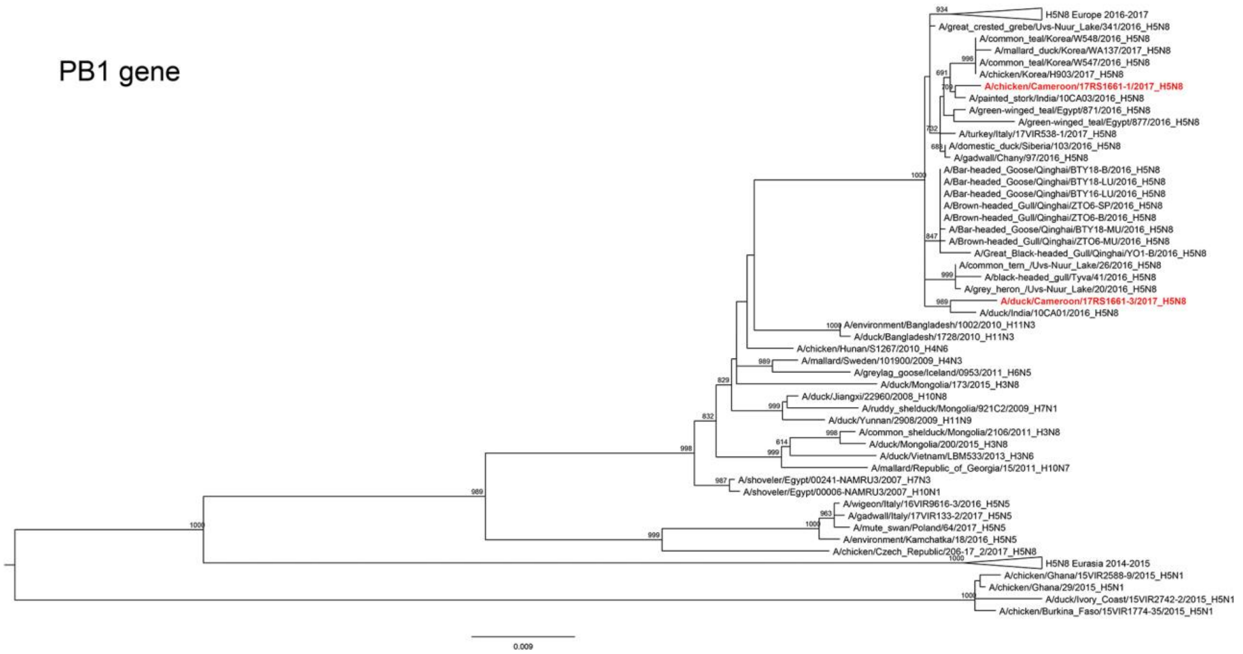

Technical Appendix 1 Figure 7. Maximum likelihood phylogenetic tree of the polymerase basic protein 1 (PB1) gene. The H5N8 viruses from Cameroon are marked in red. Bootstrap supports higher than 600/1000 are indicated next to the nodes; scale bar indicates numbers of nucleotide substitutions per site.

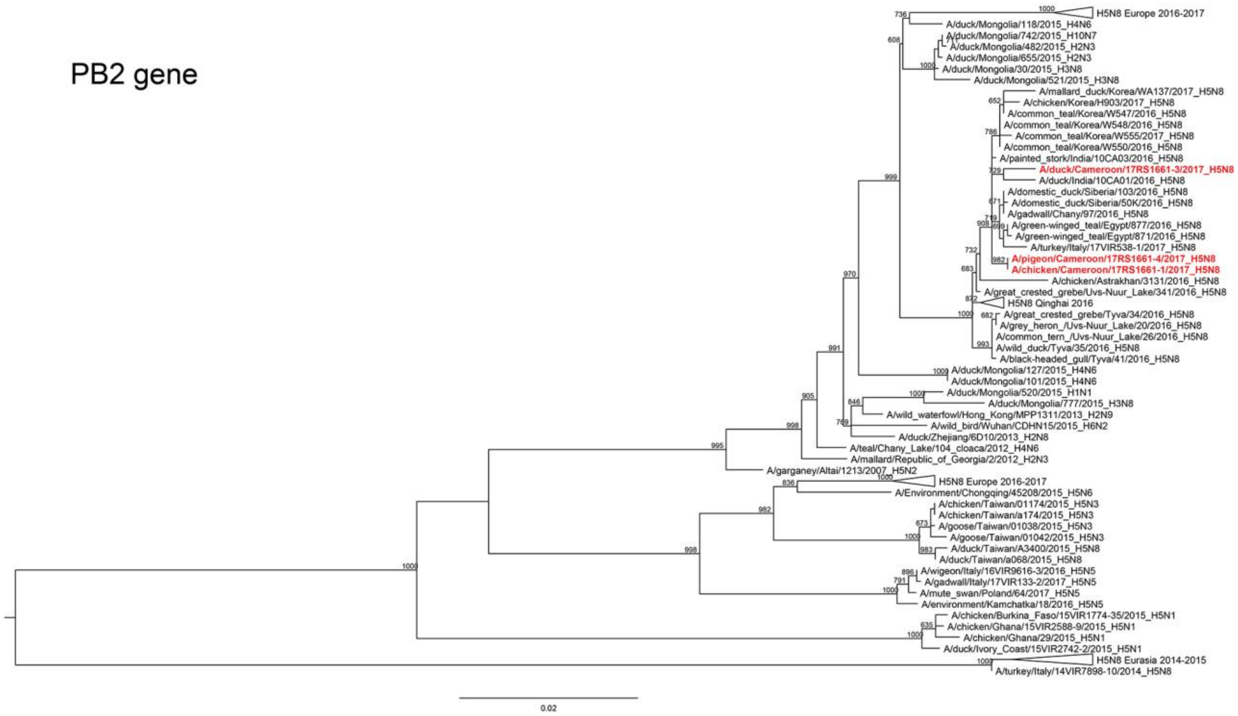

Technical Appendix 1 Figure 8. Maximum likelihood phylogenetic tree of the polymerase basic protein 2 (PB2) gene. The H5N8 viruses from Cameroon are marked in red. Bootstrap supports higher than 600/1000 are indicated next to the nodes; scale bar indicates numbers of nucleotide substitutions per site.

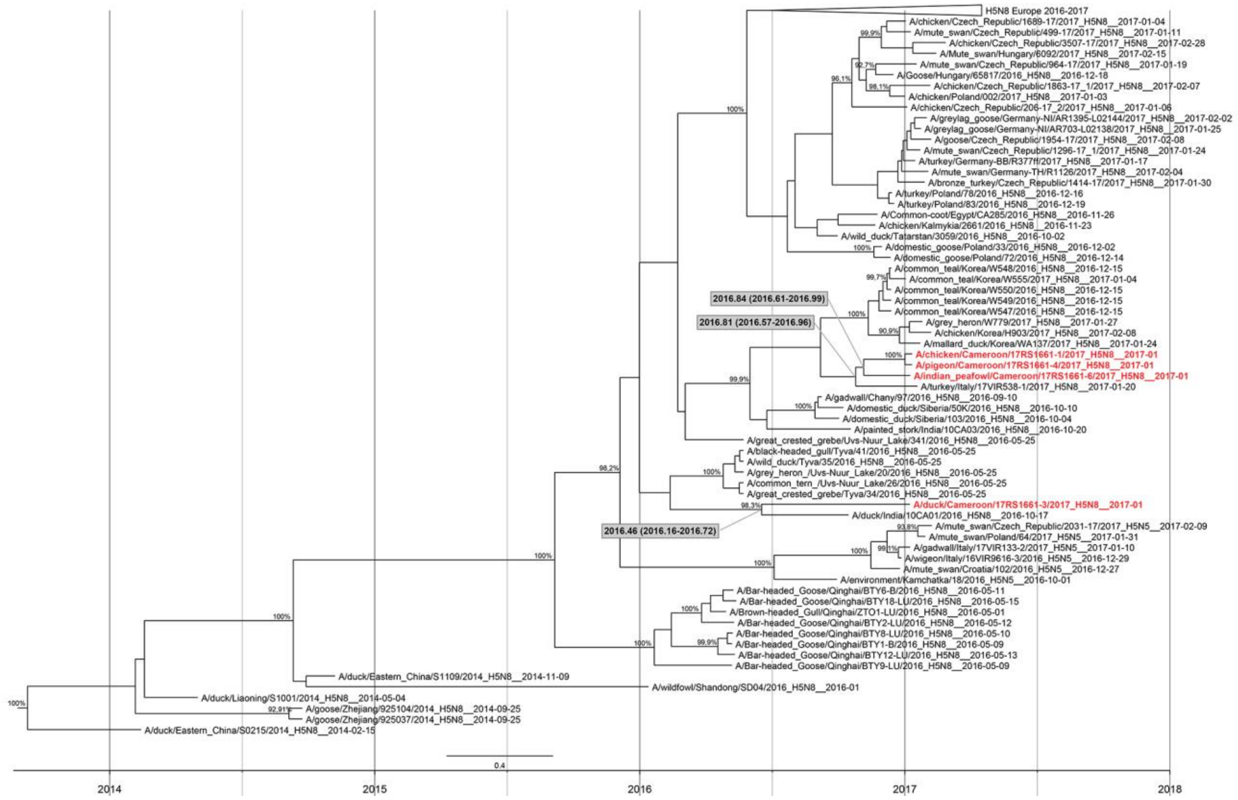

Technical Appendix 1 Figure 9. Maximum clade credibility phylogenetic tree of the hemagglutinin (HA) gene. The H5N8 viruses from Cameroon are marked in red. Posterior probability values higher than 90% are indicated next to the nodes. The mean time to the most recent common ancestor (tMRCA) and the 95% highest posterior density intervals of the relevant nodes are indicated in the gray boxes.
